# Coding-Complete Genome Sequence of a Human Respirovirus 1 Strain from a Clinical Sample in Arizona

**DOI:** 10.1128/MRA.00465-20

**Published:** 2020-06-11

**Authors:** Matthew Scotch, Rolf U. Halden, Anthony Denton, Helen Sandrolini, Rafaela S. Fontenele, Arvind Varsani

**Affiliations:** a Biodesign Center for Environmental Health Engineering, Biodesign Institute, Arizona State University, Tempe, Arizona, USA; b College of Health Solutions, Arizona State University, Phoenix, Arizona, USA; c School of Sustainable Engineering and the Built Environment, Arizona State University, Tempe, Arizona, USA; d ASU Health Services, Arizona State University, Tempe, Arizona, USA; e Biodesign Center for Fundamental and Applied Microbiomics, Biodesign Institute, Arizona State University, Tempe, Arizona, USA; f School of Life Sciences, Tempe, Arizona, USA; g OneWaterOneHealth, Arizona State University Foundation, Tempe, Arizona, USA; h AquaVitas, LLC, Scottsdale, Arizona, USA; KU Leuven

## Abstract

Human respirovirus 1 is a single-stranded, negative-sense RNA virus in the family *Paramyxoviridae*. Using a high-throughput metagenomic approach, we identified and sequenced the coding-complete genome of a human respirovirus 1 strain from a nasal pharyngeal swab sample from a local health clinic in Tempe, Arizona.

## ANNOUNCEMENT

Human respirovirus 1 (previously human parainfluenza virus 1) is a single-stranded, negative-sense RNA virus in the family *Paramyxoviridae* (genus *Respirovirus*) that causes infections and diseases (e.g., croup) in humans, mainly children (1, 2). The genus *Respirovirus* includes members with genomes of ∼15,500 nucleotides (nt) long, such as human respirovirus 1, human respirovirus 3, bovine respirovirus 3, caprine respirovirus 3, murine respirovirus, and porcine respirovirus 1 (3). In particular, human respirovirus 1 is not well represented in GenBank (4), with <800 sequence entries to date, only 91 of which are full-length or near-full-length genomes (>12,000 nt). The human respirovirus identified in this study came from an Arizona clinical sample and contains the typical genes found in other respiroviruses, i.e., matrix protein (1,047 nt), nucleocapsid protein (1,575 nt), fusion glycoprotein (1,668 nt), HN glycoprotein (1,743 nt), phosphoprotein (1,803 nt), and RNA-dependent RNA polymerase (6,693 nt).

As part of a long-term on-campus influenza surveillance study (5), influenza virus study nasal pharyngeal swab samples from a university health clinic are regularly tested for influenza types A and B, if patients present with influenza-like illness. A sample that tested negative for seasonal influenza types A and B via rapid lateral flow immunoassay (Abbott BinaxNOW) in the health clinic was analyzed using a viral metagenomic approach. One hundred microliters of the buffer from the assay was used to extract viral RNA using the High Pure viral RNA kit (Roche Diagnostics, USA). The resulting RNA was used to prepare a library using the TruSeq stranded total RNA LT sample preparation kit with Ribo-Zero human/mouse/rat (Illumina, USA). The library was sequenced on an Illumina HiSeq X Ten sequencer at Psomagen, Inc. (USA). The raw paired-end (2 × 150-bp) reads (3,496,700 bp) were trimmed using Trimmomatic v0.39 (6) with default settings and *de novo* assembled using metaSPAdes v3.14.0 (7) with k values of 33, 55, and 77. In the resulting 54,824 contigs, a 15,554-nt contig (GC content, 37%), missing ∼59 nt at the 5′ end and ∼21 nt at the 3′ end, was identified. It shares >81% genomewide pairwise identity with human respiroviruses, as determined using SDT v1.2 (8). A total of 27,066 reads mapped back to the contig with an average depth of coverage of 91×, as determined using BBMap (https://sourceforge.net/projects/bbmap). Open reading frames were identified using ORFfinder (https://www.ncbi.nlm.nih.gov/orffinder).

**FIG 1:**
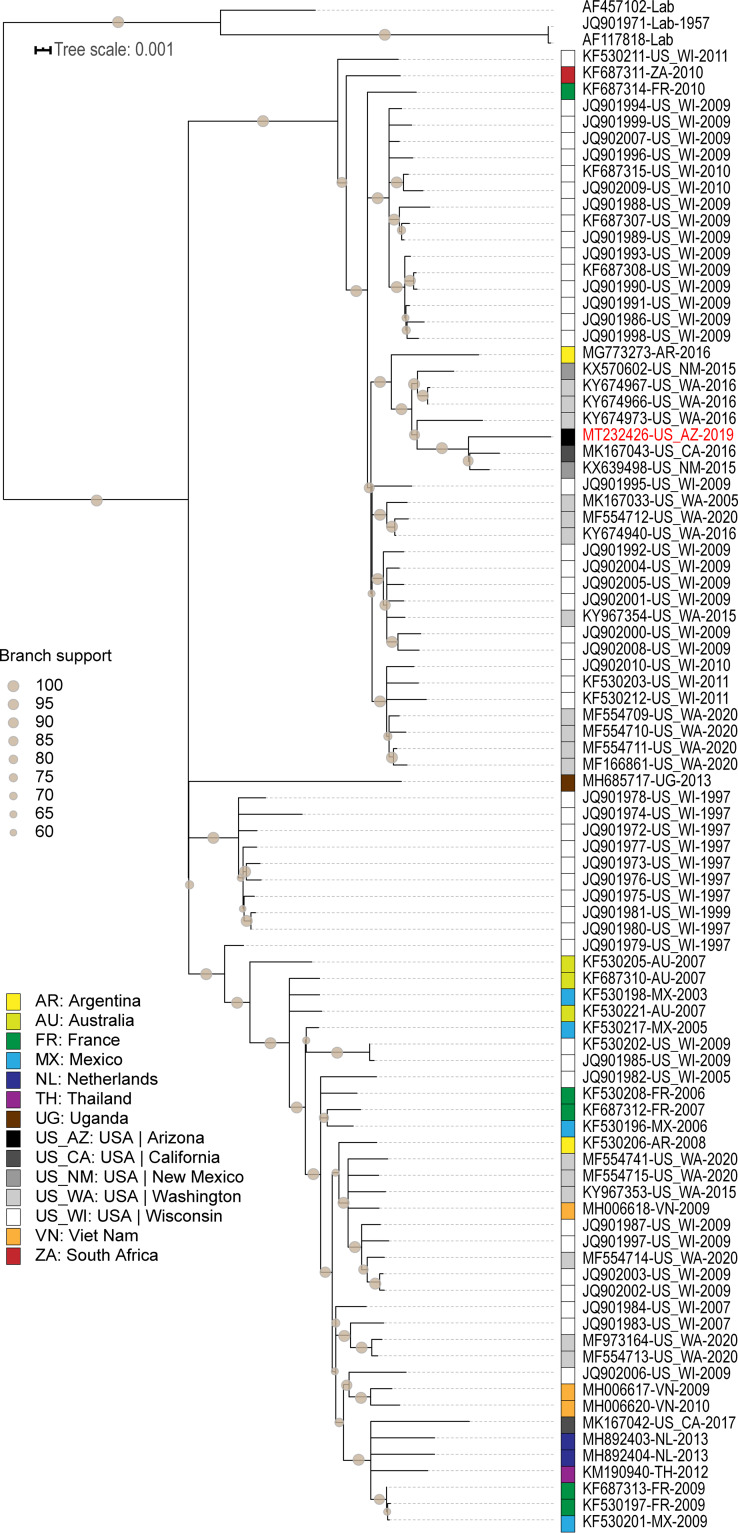
Maximum likelihood tree, with 1,000 bootstrap replicates, of human respirovirus genomes (*n* = 93) visualized and annotated using iTOL v4 (13). Branches with less than 60% bootstrap support were collapsed using TreeGraph 2 (14). The locations of sampling are color-coded, and the years of sampling are provided along with taxon names and GenBank accession numbers. The human respirovirus 1 from Arizona (USA) is highlighted in red.

A data set of 92 human respirovirus sequences of >6,500 nt available in GenBank was aligned with the sequence we identified using MUSCLE (9). The resulting alignment was used to infer a maximum likelihood phylogenetic tree using PhyML v3 (10) with GTR+G as the best substitution model selected using jModelTest (11), with 1,000 bootstrap replicates. Three non-wild-type sequences were used as an outgroup. The remaining viruses form two large clades and one smaller monophyletic clade of Wisconsin viruses. Despite a large sampling bias from a study that focused exclusively on Wisconsin (12), the Arizona human respirovirus sequence (GenBank accession number MT232426) clusters with sequences of respiroviruses from New Mexico (KX639498) and California (MK167043), sharing 99.1 to 99.2% genomewide pairwise identity (Fig. 1). Improved sampling across geographic areas is needed to study phylodynamics and the relationship between human respirovirus 1 evolution and geographic spread. In addition, linking clinical phenotype data to genomic epidemiology would allow for a better understanding of the relationship between viral genetics and clinical outcomes.

### Data availability.

Our virus sequence has been deposited in the NCBI databases SRA, under the accession number SRR11676238, and GenBank, under the accession number MT232426.

## References

[1] Ishiguro T, Kobayashi Y, Takano K, Ozawa R, Shimizu Y, Takayanagi N 2020 Two cases of primary human parainfluenza virus 1 pneumonia in which bronchoalveolar lavage fluid yielded human parainfluenza virus 1. Intern Med 59:101–105. doi:10.2169/internalmedicine.3435-19. 31511487 PMC6995725

[2] CDC. 2020 Human parainfluenza viruses (HPIVs): symptoms and illnesses. https://www.cdc.gov/parainfluenza/about/symptoms.html. Accessed 16 March 2020.

[3] Phan MVT, Arron G, GeurtsvanKessel CH, Huisman RC, Molenkamp R, Koopmans MPG, Cotten M 2019 Complete genome characterization of eight human parainfluenza viruses from the Netherlands. Microbiol Resour Announc 8:e00125-19. doi:10.1128/MRA.00125-19. 30975805 PMC6460028

[4] Sayers EW, Cavanaugh M, Clark K, Ostell J, Pruitt KD, Karsch-Mizrachi I 2020 GenBank. Nucleic Acids Res 48:D84–D86. doi:10.1093/nar/gkz956. 31665464 PMC7145611

[5] NIH. 2020 Project Information: bioinformatics framework for wastewater-based surveillance of infectious diseases on NIH. https://projectreporter.nih.gov/project_info_description.cfm?aid=9766015&icde=49788338. Accessed 30 April 2020.

[6] Bolger AM, Lohse M, Usadel B 2014 Trimmomatic: a flexible trimmer for Illumina sequence data. Bioinformatics 30:2114–2120. doi:10.1093/bioinformatics/btu170. 24695404 PMC4103590

[7] Bankevich A, Nurk S, Antipov D, Gurevich AA, Dvorkin M, Kulikov AS, Lesin VM, Nikolenko SI, Pham S, Prjibelski AD, Pyshkin AV, Sirotkin AV, Vyahhi N, Tesler G, Alekseyev MA, Pevzner PA 2012 SPAdes: a new genome assembly algorithm and its applications to single-cell sequencing. J Comput Biol 19:455–477. doi:10.1089/cmb.2012.0021. 22506599 PMC3342519

[8] Muhire BM, Varsani A, Martin DP 2014 SDT: a virus classification tool based on pairwise sequence alignment and identity calculation. PLoS One 9:e108277. doi:10.1371/journal.pone.0108277. 25259891 PMC4178126

[9] Edgar RC 2004 MUSCLE: multiple sequence alignment with high accuracy and high throughput. Nucleic Acids Res 32:1792–1797. doi:10.1093/nar/gkh340. 15034147 PMC390337

[10] Guindon S, Dufayard JF, Lefort V, Anisimova M, Hordijk W, Gascuel O 2010 New algorithms and methods to estimate maximum-likelihood phylogenies: assessing the performance of PhyML 3.0. Syst Biol 59:307–321. doi:10.1093/sysbio/syq010. 20525638

[11] Darriba D, Taboada GL, Doallo R, Posada D 2012 jModelTest 2: more models, new heuristics and parallel computing. Nat Methods 9:772. doi:10.1038/nmeth.2109. PMC4594756 22847109

[12] Beck ET, He J, Nelson MI, Bose ME, Fan J, Kumar S, Henrickson KJ 2012 Genome sequencing and phylogenetic analysis of 39 human parainfluenza virus type 1 strains isolated from 1997–2010. PLoS One 7:e46048. doi:10.1371/journal.pone.0046048. 23029382 PMC3459887

[13] Letunic I, Bork P 2019 Interactive Tree of Life (iTOL) v4: recent updates and new developments. Nucleic Acids Res 47:W256–W259. doi:10.1093/nar/gkz239. 30931475 PMC6602468

[14] Stover BC, Muller KF 2010 TreeGraph 2: combining and visualizing evidence from different phylogenetic analyses. BMC Bioinformatics 11:7. doi:10.1186/1471-2105-11-7. 20051126 PMC2806359

